# The clinical implications of sunitinib-induced hypothyroidism: a prospective evaluation

**DOI:** 10.1038/sj.bjc.6604497

**Published:** 2008-07-29

**Authors:** P Wolter, C Stefan, B Decallonne, H Dumez, M Bex, P Carmeliet, P Schöffski

**Affiliations:** 1 Department of General Medical Oncology, University Hospital Gasthuisberg, Catholic University Leuven, Leuven Cancer Institute, Herestraat 49, Leuven B-3000, Belgium; 2 Department of Endocrinology, University Hospital Gasthuisberg, Catholic University Leuven, Leuven Cancer Institute, Herestraat 49, Leuven B-3000, Belgium; 3 Center for Transgene Technology and Gene Therapy, K.U. Leuven, Leuven B-3000, Belgium; 4 Department for Transgene Technology and Gene Therapy, VIB Leuven, Leuven B-3000, Belgium

**Keywords:** sunitinib, prospective study, hypothyroidism, gastrointestinal stromal tumour, renal cell carcinoma

## Abstract

Sunitinib is approved for the treatment of metastatic renal cell carcinoma (RCC) and imatinib-resistant or -intolerant gastrointestinal stromal tumours (GIST). Several studies have identified unexpected rates of thyroid dysfunction with sunitinib treatment. We performed a prospective observational study with the aim of more accurately defining the incidence and severity of hypothyroidism in RCC or GIST patients receiving sunitinib. Thyroid function was assessed at baseline and on days 1 and 28 of each treatment cycle. Thyroid antibodies were assessed at baseline and during follow-up if abnormal thyroid function tests were recorded. Sixteen patients (27%) developed sub- or clinical hypothyroidism and required hormone replacement and 20 patients (34%) showed at least one elevated thyroid-stimulating hormone not requiring therapeutic intervention. Twenty patients (34%) did not develop any biochemical thyroid abnormality. Thus, sunitinib can induce (sub-) clinical hypothyroidism, warranting close monitoring of thyroid function. We propose a new algorithm for managing this side effect in clinical practise.

Sunitinib (SUTENT; Pfizer Inc., New York NY, USA) is an oral multitargeted tyrosine kinase inhibitor with both anti-angiogenic and anti-tumor activities mediated by signal blockade of several receptors, including VEGFR-1, -2 and -3; KIT; PDGFR-*α* and PDGFR-*β*; FLT-3; CSF-1 and RET (Chow and Eckhardt, 2007; Roskoski Jr 2007). Its efficacy in metastatic renal cell carcinoma (RCC) (Motzer *et al*, 2006) and in imatinib-resistant or -intolerant gastrointestinal stromal tumours (GIST) (Demetri *et al*, 2006) led to Food and Drug Administration (FDA) and European Medicines Agency (EMEA) approval for treatment of these cancers (Goodman *et al*, 2007; Rock *et al*, 2007). Sunitinib may also be beneficial in other malignancies, including neuroendocrine, breast, colon and non-small-cell lung cancer (Saltz *et al*, 2007; Burstein *et al*, 2008; Socinski *et al*, 2008).

Our group has gained clinical experience with sunitinib administration, primarily in patients with RCC and GIST (Schöffski *et al*, 2006a, 2006b; Wolter *et al*, 2007), since 2005. Having first identified sporadic cases of thyroid dysfunction during sunitinib treatment, we evaluated this phenomenon in a limited number of patients, both retrospectively (*n*=14) and prospectively (*n*=19) (Schöffski *et al*, 2006b). In both studies, we found an unexpected high frequency of hypothyroidism (57 and 37%, respectively). This uncommon drug-induced side effect was not previously identified in clinical trials, in which sunitinib toxicities included fatigue, nausea, vomiting, hand-foot-skin reaction, rash, hypertension and/or diarrhoea (Demetri *et al*, 2006; Faivre *et al*, 2006; Motzer *et al*, 2006). An association between sunitinib treatment and thyroid dysfunction has been also described by other groups (Desai *et al*, 2006; Martorella *et al*, 2006; Shaheen *et al*, 2006; Mannavola *et al*, 2007; Rini *et al*, 2007; Wong *et al*, 2007). However, the previous studies were limited by factors related to study design including variable definition of hypothyroidism, incomplete panel of thyroid function tests (TFTs) or lack of measurements for all patients and inclusion of patients with underlying thyroid disorders.

Here, we present the results of our prospective evaluation of incidence and severity of the new-onset hypothyroidism in a larger number of sunitinib-treated cancer patients (*n*=59). We include clearly defined exclusion criteria, baseline thyroid-stimulating hormone (TSH) values in all patients and thyroid antibodies in the majority of patients, as well as systematically evaluating TFTs during treatment. Furthermore, we discuss comparatively our study with the currently available data on sunitinib-induced thyroid dysfunction, and propose for the first time an algorithm for routine treatment of this clinical phenomenon.

## Patients and methods

### Patients and treatment schedule

Our single-centre, prospective, observational study included patients receiving sunitinib in the Department of General Medical Oncology at the University Hospital Gasthuisberg (Leuven, Belgium). Between November 2005 and June 2007, a total of 74 patients received sunitinib for metastatic, immunotherapy-resistant RCC or imatinib-refractory or -intolerant GIST. Patients with abnormal TFTs at baseline (before sunitinib initiation), with previous thyroid hormone replacement due to underlying thyroid disease or under sunitinib treatment for less than 4 weeks, were excluded from the study. Patients had no food restriction, and medication other than sunitinib was not standardised. Sunitinib 50 mg day^−1^ was administered orally for 4 weeks, followed by a 2-week rest period. Doses were adjusted based on haematological and non-haematological adverse events, according to the manufacturer's recommendations. Study end points included the incidence and severity of sunitinib-induced thyroid dysfunction.

### Evaluation of thyroid function

All patients had thyroid function assessed at baseline and on days 1 and 28 of each treatment cycle, which was determined by serum TSH, total triiodotyronine (T3) and the free thyroxine index (FTI) calculated from total thyroxine (T4) and the residual T-uptake. Antibodies against thyroid peroxidase (TPOAb), thyroglobulin (TgAb) and the TSH receptor (TR-Ab) were also assessed at baseline in most patients and during follow-up if abnormal TFTs were recorded. Reverse T3 and Tg were also measured in patients with abnormal TFTs. Serum TSH, total T3, T4 and T-uptake were measured by an electrochemiluminescent immunoassay (ECLIA; Roche, Mannheim, Germany); TPOAb and TgAb by a radioligand assay (Brahms, Henningsdorf, Germany); and TR-Ab by a radio receptor assay (Brahms). Serum samples were collected, handled and analysed according to internal standard operating procedures.

Our laboratory reference ranges are 0.27–4.20 mIU l^−1^ for TSH; 5.1–14.1 *μ*g dl^−1^ for total T4; 80–200 ng dl^−1^ for total T3; 4.8–12.7 for FTI; and 80–130% for T-uptake. The cutoff level for TPOAb is 100 U ml^−1^. Antibodies against thyroglobulin >200 U ml^−1^ is considered positive. Antibodies against thyroglobulin <1 IU l- is considered negative and >1.5 IU l^−1^ as positive.

### Definition of hypothyroidism and hyperthyroidism

The biochemical diagnosis of subclinical hypothyroidism and hyperthyroidism was determined in accordance with guidelines of the American Thyroid Association (ATA), the American Association of Clinical Endocrinologists (AACE) and the Endocrine Society (Surks *et al*, 1990, 2004). Subclinical hypothyroidism is considered as serum TSH above the upper limit of normal (ULN=4.20 mIU l^−1^ in our laboratory), with FTI within normal limits. Clinical hypothyroidism is defined as low serum FTI together with elevated TSH. Subclinical hyperthyroidism is defined as serum TSH below the lower limit of normal (LLN=0.27 mIU l^−1^ in our laboratory), with serum T4 and T3 within normal ranges. Overt hyperthyroidism is considered as low TSH and elevated FTI.

Patients with overt hypothyroidism and those with at least two consecutive TSH measurements >10 mIU l^−1^ and symptoms compatible with hypothyroidism (e.g., fatigue, cold intolerance, constipation or weight gain) received thyroid hormone replacement therapy with L-thyroxine. Target TSH concentration for replacement intervention was 0.5–2 mIU l^−1^. All patients with overt hyperthyroidism and symptoms compatible with hyperthyroidism (e.g., anxiety, weakness, tremor, palpitations, heat intolerance, increased perspiration and weight loss, despite a normal or increased appetite and diarrhoea) were treated.

## Results

### Patient characteristics

In total, 59 out of 74 patients were eligible for evaluation. Of the 15 patients who were excluded from the study, 5 patients had abnormal baseline TFTs, 4 patients were already receiving thyroid hormone replacement due to underlying thyroid disease and 6 patients received sunitinib treatment for less than 4 weeks. The percentage of patients with abnormal baseline TFTs (7%) agrees with the expected prevalence of thyroid dysfunction in the general population. Demographics, disease and treatment characteristics of the 59 eligible patients are presented in Table 1. Almost two-thirds of eligible patients had RCC (*n*=42), with the majority having clear cell histology, with the remainder belonging to the GIST cohort (*n*=17). The patient median age/range and gender ratio was similar for the two cohorts. Prior treatment included interleukin-2 and/or interferon-α for the RCC group and imatinib for the GIST group. In the total study population (*n*=59), median TSH at baseline was 1.47 mIU l^−1^ (range 0.28–3.94), which was within our laboratory reference range. Overall, median treatment with sunitinib was 29 weeks (range 4–82), with a slightly more prolonged median follow-up of 34 weeks (range 4–88). Haematological or non-haematological grade III and IV toxicities were observed in 18 (43%) RCC and 6 (35%) GIST patients.

### Thyroid function during sunitinib administration

Table 2 provides a summary of TFT results in both RCC and GIST cohorts during sunitinib treatment. We followed ATA, AACE and the Endocrine Society guidelines to establish a biochemical diagnosis of subclinical hypothyroidism and hyperthyroidism (Surks *et al*, 1990, 2004). Although there is some controversy as to whether the TSH ULN should be reduced, we considered TSH >4.20 mIU l^−1^ to be elevated. Only 20 patients (34%) had no biochemical thyroid abnormality. Twenty patients (34%) showed at least one elevated TSH not requiring therapeutic intervention and 16 (27%) patients developed sub- or clinical hypothyroidism requiring treatment. In three patients (5%) at least one serum TSH fell below the LLN (LLN=0.27 mIU l^−1^), with serum T4 and T3 within normal ranges.

We observed TSH levels of up to 120 mIU l^−1^, approximately 30-fold ULN. Thyroid-stimulating hormone elevation can be quite substantial even within one sunitinib cycle, as observed, for instance, in one patient, from a normal 0.19 to a high of 78.63 mIU l^−1^. In most patients, such high levels of TSH were accompanied by typical features of hypothyroidism, such as progressive fatigue, myalgia, cold intolerance and constipation. We did not encounter myxoedema coma cases, but such cases may exist, as demonstrated by the study of Mannavola *et al* (2007).

**Figure 1:**
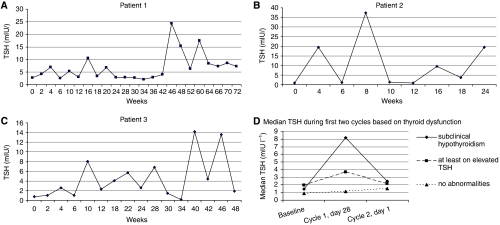
Representative courses of TSH (mIU l^−1^) in three patients receiving sunitinib (**A**–**C**); median TSH during first cycles of sunitinib treatment based on severity of thyroid dysfunction (**D**).

Thyroid function abnormalities were detected relatively early during treatment, with median time to abnormal TSH of 4 weeks (range 2–46). Representative courses of TSH levels in three patients treated with sunitinib are presented in Figure 1A–C. Note the characteristic alternate course of TSH concentrations (zigzag shape), with normal levels at baseline, elevated on day 28 of a 4-week treatment cycle, and normalised at the end of the 2-week rest period. In patients experiencing TFT abnormalities, such pattern was observed during most cycles of sunitinib treatment. In the 16 patients developing (sub)clinical hypothyroidism requiring hormone replacement, median TSH at baseline was 1.45 mIU l^−1^ (range 0.59–3.94), increased to a median of 8.15 mIU l^−1^ (range 2.61–26.32) by day 28 of the first cycle, and decreased to a median of 2.5 mIU l^−1^ (range 0.92–13.23) by day 1 of the second cycle. A similar pattern was also found in the subgroup of patients with at least one elevated TSH without requiring treatment; however, the increase in TSH was more moderate. Thus, for these patients, median TSH at baseline was 1.96 mIU l^−1^ (range 0.78–3.9), rose by day 28 of the first sunitinib cycle to 3.65 mIU l^−1^ (range 1.15–7.22), and dropped by day 1 of the second cycle to a median of 2.14 mIU l^−1^ (range 0.52–4.08) (Figure 1D).

The median duration of sunitinib treatment in the group developing hypothyroidism was 48 weeks (range 4–81), which is much longer than in the group with no thyroid function abnormality (21 weeks; range 4–82). The percentage of patients with (sub)clinical hypothyroidism requiring treatment was higher in the RCC group (33%) than in the GIST group (12%). Dose reductions for grades III–IV haematological and non-haematological toxicities were more frequent in the hypothyroid group than in the normal thyroid group.

Subclinical hypothyroidism was preceded by a short period of low TSH and elevated T3/T4 in 4 out of the 16 patients developing hypothyroidism. Although TPOAb was negative in all four patients, ultrasound revealed thyroid hypovascularity and serum Tg was elevated in 2, suggesting underlying drug-induced thyroiditis.

Low titres of TPOAb were found in only 2 (4%) out of 49 total evaluable patients, whereas TgAb and TR-Ab were positive in 2 and 1 patients, respectively.

## Discussion

Our study prospectively analysed the occurrence and severity of hypothyroidism in cancer patients receiving sunitinib. Our patient population comprises 59 patients, the majority of whom had metastatic cytokine-resistant RCC, and the rest had imatinib-resistant or -intolerant GIST. Although our study confirms that sunitinib may induce biochemical and clinical hypothyroidism, it adds several features compared with previously published studies (Table 3). The definition of hypothyroidism was somewhat variable in previous studies. Other limitations include lack of complete TFTs before or during treatment, and/or measurements available in some but not all patients. When our manuscript was in preparation, Mannavola *et al* published results from a prospective evaluation, involving a smaller number of patients (*n*=24) (Mannavola *et al*, 2007).

Following initiation of sunitinib, one-third of patients did not develop any biochemical TFT abnormalities. We observed a transient elevation of TSH in 34% of cases and (sub)clinical primary hypothyroidism requiring treatment in a further 27% of patients. As all patients had normal thyroid function at baseline, the latter percentage applies to the new-onset hypothyroidism. The frequency of (sub)clinical hypothyroidism in sunitinib-treated patients varies substantially among the reported studies (Table 3), but is up to 10-fold higher than the occurrence of hypothyroidism in the general population (Laurberg *et al*, 2005; Lazarus, 2007).

Hypothyroidism is uncommon in the general population, with a prevalence of 4–8.5% (Surks *et al*, 1990, 2004). Reliable data on hypothyroidism prevalence in cancer patients are not available; however, some preliminary data suggest a slightly higher frequency in some tumour types such as melanoma and breast cancer (Ellerhorst *et al*, 2003; Jiskra *et al*, 2004; Shah *et al*, 2006).

In general, hypothyroidism is particularly common in older age groups and women (Laurberg *et al*, 2005). However, in our study, the median patient age was 61 years (range 42–77) and male : female ratio was 3 : 1, indicating that these patient characteristics do not contribute substantially to the observed incidence in our patients. Similar patient characteristics were also found in the study by Rini *et al* (2007).

On the basis of initial study protocol, we started hormone replacement therapy in patients with persistent TSH (>10 mIU l^−1^ on day 1 of two consecutive cycles) showing typical symptoms of hypothyroidism; symptoms resolved in the majority of cases but not all. The clinical presentation of patients with hypothyroidism is highly variable and non-specific, and side effects of sunitinib can be very similar to symptoms of hypothyroidism, certainly in patients with advanced cancer. Therefore, we cannot be sure whether fatigue in sunitinib-treated patients can be exclusively explained by primary hypothyroidism. The hypothesis of Garfield *et al* (2007) that hypothyroidism may be associated with improved outcome in certain types of cancer warrants further investigation, but hormone replacement therapy should not be withheld if clinically indicated (Garfield *et al*, 2007).

Patients with preexisting thyroid function abnormalities may require higher doses of hormone replacement therapy to maintain an euthyroid state while treated with sunitinib. In our centre, TSH levels increased in three patients with a history of well-controlled hypothyroidism before sunitinib treatment, and adjustment of hormone replacement therapy was necessary. These patients were excluded from the prospective evaluation, however, a similar finding was reported by Rini *et al* (2007). More recently, De Groot *et al* (2006) reported the case of a GIST patient who was treated simultaneously with sunitinib and L-thyroxine following previous thyroidectomy and ^131^I-ablation due to follicular thyroid carcinoma (de Groot *et al*, 2006). The patient developed overt hypothyroidism while receiving sunitinib, and L-thyroxine doses were increased.

The most common cause of hypothyroidism in the general population is chronic autoimmune (Hashimoto's) thyroiditis, resulting in antibody-mediated destruction of the thyroid tissue (Laurberg *et al*, 2005). More than 90% of such patients have elevated serum TPO or Tg antibodies. Desai *et al* (2006) reported TPOAb in 2 out of 42 patients, for whom values were normal (Desai *et al*, 2006). Rini *et al* (2007) measured TgAb (13 abnormal values of 44 available from 66 enrolled patients) and found no correlation between the presence of antibodies and either incidence or severity of other TFT abnormalities (Rini *et al*, 2007). Accordingly, in our patients, anti-TPO or anti-Tg antibodies were elevated only in a minority of those with elevated TSH. Similarly, in the study of Mannavola *et al*, TPOAb remained negative during all cycles in all patients who had normal TFTs at baseline (Mannavola *et al*, 2007). These findings do not support an underlying autoimmune process for developing hypothyroidism in sunitinib-treated patients.

Mild thyrotoxicosis may, however, precede hypothyroidism in some but not all patients. Desai *et al* (2006) identified one or more TSH concentrations <0.5 mIU l^−1^, thus indicating the development of subclinical hyperthyroidism before hypothyroidism (Desai *et al*, 2006). In our prospective study, we identified four such cases, suggesting that drug-induced thyroiditis causing leakage of thyroid hormone into the circulation can potentially be an underlying mechanism for sunitinib-induced hypothyroidism.

During sunitinib therapy, most of our patients underwent repeated CT scan with intravenous iodinated contrast, every 6–8 weeks. Iodine-containing contrast media can transiently elevate serum TSH, but not free T3 and T4 (Gartner and Weissel, 2004). Although the effect of repeated exposure to iodine on TSH is difficult to estimate in our patients, it should be noted that its level increased already soon after starting sunitinib administration in our RCC cohort.

Seriously ill patients often show abnormal TFTs not necessarily associated with thyroid dysfunction, the so-called ‘non-thyroidal illness (NTI)’ or ‘low T3 syndrome’ (Demers, 2004). Therefore, peripheral thyroid hormone levels should be interpreted with caution in these patients. Serum TSH concentrations remain within reference limits for most NTI patients, although occasionally a mild transient TSH decrease can be observed at the beginning of NTI, followed by a rebound to mildly elevated values during recovery. Thus, NTI cannot be excluded as a confounding factor in some patients and may result in a slight overestimation of the percentage of patients developing slight biochemical TFT abnormalities. In the two other groups defined as ‘(sub)clinical hypo-/hyperthyroidism requiring treatment’, thyroid abnormalities had to be persistent and/or associated with clinical symptoms, which is not compatible with NTI.

Primary hypothyroidism is not a common complication of therapeutic drugs. Among the drugs known to affect thyroid function are lithium, thioamides, amiodarone and cytokines, such as interferon and interleukin-2. However, the mechanisms are not well understood and may also differ from that of sunitinib-induced hypothyroidism. In our population, most patients with metastatic RCC had received cytokine therapy before starting sunitinib. We cannot exclude that this may have influenced the course of hypothyroidism. It may also explain the difference in thyroid dysfunction between the RCC (33%) and the GIST (12%) groups, as the latter did not have to receive cytokine therapy before sunitinib, and imatinib is not known to induce thyroid dysfunction.

According to our study, there is no clear association between the daily dose of sunitinib and developing thyroid dysfunction. Thus, in contrast to the patient group with normal thyroid function during treatment, patients developing hypothyroidism underwent, for the most part, one or two dose reductions for grades III–IV haematological and non-haematological toxicities, suggesting that hypothyroidism in these patients reflects a particular susceptibility to sunitinib rather than dose dependency.

The molecular mechanisms of sunitinib-induced hypothyroidism are currently unknown. Autoimmune-mediated hypothyroidism could not be demonstrated as an aetiological factor in this study, which is in accordance with the results of other groups. Sunitinib may have a direct effect on the thyroid, for example, through the inhibition of VEGFR and/or PDGFR. More recent studies in a mouse model have shown that VEGFR inhibition can induce capillary regression in various organs, including thyroid. Moreover, the vasculature of the thyroid had the greatest regression of all organs (Baffert *et al*, 2006; Kamba *et al*, 2006). Remarkably, in this animal model, thyroid capillaries regenerated in the absence of VEGFR inhibition. Such a response may explain the rhythmic pattern of TSH seen in patients treated with the 4/2 schedule. It is interesting that in the studies of Baffert *et al* (2006) and Kamba *et al* (2006), TSH also increased in mice treated with VEGF inhibition. Sunitinib may also inhibit TPO activity leading to reduced synthesis of the thyroid hormones, as suggested by *in vitro* studies (Wong *et al*, 2007). Indirectly, sunitinib may affect the thyroid by interfering with the metabolism of T4/T3 hormones, or with thyroid hormone action at the pituitary level. In patients treated with sorafenib, another tyrosine kinase inhibitor, thyroid dysfunction seems not to be a frequent side effect (Tamaskar *et al*, 2008; Wolter *et al*, manuscript in preparation).

**Figure 2:**
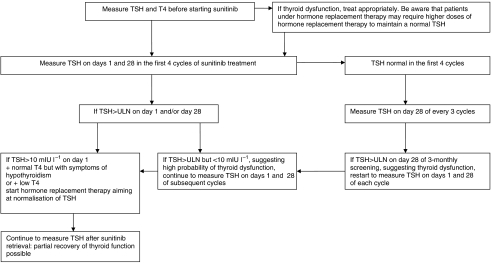
Proposed algorithm to diagnose and treat thyroid dysfunction during sunitinib treatment.

To address the clinical management of thyroid dysfunction in patients treated with sunitinib, we suggest an algorithm for TFT monitoring and intervention (Figure 2). In our study, patients who developed (sub)clinical hypothyroidism requiring treatment (*n*=16) showed TSH elevation early, within median 4 weeks, following sunitinib initiation. By contrast, some previously published studies concluded that thyroid dysfunction can occur later during sunitinib treatment (Table 3). However, these studies are mainly retrospective, and consistent TSH measurements on both days 1 and 28 of sunitinib cycles are lacking, which most probably account for this discrepancy (Desai *et al*, 2006; Martorella *et al*, 2006). Our observations that TFT abnormalities occur rather early after sunitinib initiation are, however, in better agreement with the prospective study by Mannavola *et al* (2007) on 24 GIST patients, where TSH measurements were performed on both days 1 and 28 and hypothyroidism developed at median of 3 cycles (range: 1–6). Moreover, we did not observe any patient with normal TFT during the first cycles of sunitinib who developed clinical hypothyroidism later in the course of treatment.

In summary, we recommend that all patients treated with sunitinib have TFTs performed on days 1 and 28 of the first four cycles (Figure 2). By applying this intensive initial screening, we consider that we can detect patients at risk of developing sunitinib-induced thyroid dysfunction early following drug initiation. Moreover, on the basis of our study, monitoring of patients with no TFT abnormalities within the four first cycles can be subsequently performed more rarely, every three cycles, unless clinically indicated. In these cases, measurements are best recommended on day 28 rather than on day 1 of a cycle, as the likelihood to detect TFT abnormalities in highest at this point. Furthermore, we suggest starting hormone replacement therapy in patients with persistent TSH >10 mIU ml^−1^ and either low T4 or normal T4 but with typical symptoms of hypothyroidism. As TSH level declines and could be normalised by the end of the 2-week rest period of a sunitinib cycle, decisions to start hormone replacement should be based on TSH levels on day 1 of a new sunitinib cycle rather than on day 28 of a current cycle, to avoid overtreatment. We have encountered such a situation initially in our practise of treating sunitinib-induced thyroid dysfunction. Although during our study we initiated hormone replacement after TSH >10 mIU l^−1^ on day 1 of two consecutive cycles, we concluded for our algorithm that it is more prudent to start the therapy already after one cycle rather than waiting for an additional cycle. Although an elevated TSH at day 28 of a cycle may be associated with transient, reversible thyroid dysfunction, an elevated TSH at day 1 of a cycle, may already indicate a more pronounced damage to the thyroid, supporting our suggestion for treatment initiation at this point. Patients with preexisting thyroid function abnormalities may require higher doses of TSH to maintain an euthyroid state while treated with sunitinib. Although our data in measuring TSH after suninitib retrieval is limited, we also recommend continuing measuring TSH in such cases, as partial recovery of the thyroid function may occur. This observation is also supported by the study of Mannavola *et al* (2007).

In conclusion, it is important that clinicians consider the potential side effect of sunitinib on thyroid function. Sunitinib-induced hypothyroidism is easily manageable with hormone replacement therapy, and should not withhold the use of sunitinib when indicated for cancer treatment. Hormone replacement is necessary to reduce symptoms of hypothyroidism, such as fatigue, but also to avoid the potentially life-threatening complications of severe hypothyroidism, such as myxoedema coma.

We admit that our recommendations for thyroid management in patients under sunitinib may need further validation in a prospective randomized trial. We believe, however, that our data so far support the proposed algorithm, and will help clinicians in their daily practise until more validated guidelines become available.

## Figures and Tables

**Table 1:** Patient/treatment characteristics

**Cohort**	**RCC**	**GIST**	**RCC+GIST**
No. of patients	42	17	59
Male, *n* (%)	31 (74)	13 (76)	44 (75)
Female, *n* (%)	11 (26)	4 (24)	15 (25)
Median age at start, years (range)	61.5 (42–77)	61 (42–74)	61 (42–77)
Prior nephrectomy, *n* (%)	39 (93)	NA	39 (66)
Prior treatment, *n* (%)	39 (93)^a^	17 (100)^b^	56 (95)
			
*Histology, n (%)*			
Clear cell	38 (90)	NA	
Chromophobe	1 (2.5)		
Papillary	1 (2.5)		
Mixed	2 (5)		
Median treatment time with sunitinib, weeks (range)	29 (4–82)	33 (10–82)	29 (4–82)
Patients with sunitinib dose reductions, *n* (%)	18 (43)	6 (35)	24 (41)

GIST**=**gastrointestinal stromal tumour; NA=not applicable; RCC=renal cell carcinoma.

a IL-2/INF-*α*.

b Imatinib.

**Table 2:** Thyroid function during sunitinib treatment

**Cohort**	**RCC (*n*=42)**	**GIST (*n*=17)**	**RCC+GIST (*n*=59)**
No biochemical thyroid abnormalities, *n* (%)	11 (26)	9 (53)	20 (34)
Median time to abnormal TSH, weeks (range)	4 (2–22)	9 (4–46)	4 (2–46)
At least one ↑ TSH (no treatment required), *n* (%)	14 (33)	6 (35)	20 (34)
(Sub)clinical hypothyroidism (treatment required), *n* (%)	14 (33)	2 (12)	16 (27)
At least one ↓ TSH (no treatment required), *n* (%)	3 (7)	0	3 (5)
(Sub)clinical hyperthyroidism (treatment required), *n* (%)	0	0	0
TPOAb-positive patients	2^a^	0^b^	2

GIST**=**gastrointestinal stromal tumour; NA=not applicable; RCC=renal cell carcinoma.

a TPOAb measurements available in 38 out of 42 patients with RCC.

b TPOAb measurements available in 11 out of 17 patients with GIST.

**Table 3:** Studies evaluating sunitinib-induced hypothyroidism

**Study reference**	**Type of study**	**Tumour**	***n***	**Prior treatment**	**Baseline TSH (mIU/l), *n* (%)**	**No thyroid abnormality *n* (% of patients)**	**(Sub)clinical hypothyroidism *n* (% of patients)**	**(Sub)clinical hyperthyroidism (% of patients)**	**Thyroid-Ab**	**Median time to abnormal TSH (weeks)**
Desai *et al* (2006)	P+R Single centre	GIST	42	Imatinib	42 (100)	16 (38)	Total: 26 (62)	6 (14) (temporarily)	TPOAb: normal in 2/42	50
Rini *et al* (2007)	P+R Single centre	RCC	66	Naive: *n*=30 Cytokine: *n*=30 Bevacizumab: *n*=6	37 (56)	10 (15)	Total: 56 (85) Symptoms: 47 (71) Treated: 17 (25)	0	TgAb: abnormal in 13/44 TPOAb: no data	6
Wong *et al* (2007)	R Single centre	Solid tumours, GIST, RCC	40	NS	8 (20)	14 (35)	Total: 21 (53) Symptoms: 8 (20)	3 (8%)	NS	20
Martorella *et al* (2006)	P+R Single centre	RCC	39	IL-2: *n*=39	NS	—	8 (20)	—	Abnormal in 2/7	52
Shaheen *et al* (2006)	R Single centre	RCC	55	NS	NS	15 (27)	Total: 40 (73) Symptoms: 33 (60) Treated: 12 (22)	—	NS	NS
Mannavola *et al* (2007)	P Two centres	GIST	24	NS	24 (100)	7 (29)	10 (46) ↑ TSH: 6 (34)	—	TPOAb normal in all but one patient	16
Current study	P Single centre	GIST RCC	59	GIST: imatinib (all) RCC: IFN/IL-2 *n*=39	59 (100)	20 (34)	Total: 36 (61) Treated: 16 (27) At least one ↑ TSH: 20 (34)	—	TPOAb in 49 patients, normal in 47 patients	4

GIST**=**gastrointestinal stromal tumour; NS=not specified; P=prospective; R=retrospective; RCC=renal cell carcinoma; TSH=thyroid-stimulating hormone.
